# Texture discrimination and multi-unit recording in the rat vibrissal nerve

**DOI:** 10.1186/1471-2202-7-42

**Published:** 2006-05-23

**Authors:** Ana L Albarracín, Fernando D Farfán, Carmelo J Felice, Emilio E Décima

**Affiliations:** 1 Laboratorio de Neurociencia, Facultad de Medicina (FM), Universidad Nacional de Tucumán (UNT) – Tucumán – Argentina; 2 Departamento de Biongeniería (DBI), Facultad de Ciencias Exactas y Tecnología (FACET), Universidad Nacional de Tucumán (UNT) – Tucumán – Argentina, Also with Instituto Superior de Investigaciones Biológicas (INSIBIO), Consejo Nacional de Investigaciones Científicas y Técnicas (CONICET), Argentina

## Abstract

**Background:**

Rats distinguish objects differing in surface texture by actively moving their vibrissae. In this paper we characterized some aspects of texture sensing in anesthetized rats during active touch. We analyzed the multifiber discharge from a deep vibrissal nerve when the vibrissa sweeps materials (wood, metal, acrylic, sandpaper) having different textures. We polished these surfaces with sandpaper (P1000) to obtain close degrees of roughness and we induced vibrissal movement with two-branch facial nerve stimulation. We also consider the change in pressure against the vibrissa as a way to improve the tactile information acquisition. The signals were compared with a reference signal (control) – vibrissa sweeping the air – and were analyzed with the Root Mean Square (RMS) and the Power Spectrum Density (PSD).

**Results:**

We extracted the information about texture discrimination hidden in the population activity of one vibrissa innervation, using the RMS values and the PSD. The pressure level 3 produced the best differentiation for RMS values and it could represent the "optimum" vibrissal pressure for texture discrimination. The frequency analysis (PSD) provided information only at low-pressure levels and showed that the differences are not related to the roughness of the materials but could be related to other texture parameters.

**Conclusion:**

Our results suggest that the physical properties of different materials could be transduced by the trigeminal sensory system of rats, as are shown by amplitude and frequency changes. Likewise, varying the pressure could represent a behavioral strategy that improves the information acquisition for texture discrimination.

## Background

Rodents as well as many mammals are characterized by the presence of vibrissae or whiskers located on both sides of the muzzle [1]. During exploration, rats actively sweep their vibrissae in a rhythmic forward and backward motion [2-4]. It has been stated that rats, due to their poor vision, use this behavior to explore their environment [5].

Each vibrissa sits in a follicle innervated by a deep vibrissal nerve arising from the infraorbital branch of the trigeminal nerve [6]. Mechanoreceptors, such as Merkel cells, lanceolate terminals and free nerve endings [7], are activated during the vibrissae movements and this information travels along the trigeminal pathway. The signals enter the brain stem through trigeminal nerve cells, and progress, through the trigeminal complex and thalamus, to the barrel cortex.

Small muscles are associated with vibrissal follicles and have the form of a sling connecting two adjacent follicles of the same row [8]. Two branches of the facial nerve, the buccal branch and the upper division of the marginal mandibular branch [6], innervate follicular muscles and the contraction of these muscles produces the forward vibrissal movement.

Studies in tactile discriminative behavior agree that rats are able to learn a rough-smooth discrimination task by actively palpating the discriminanda (sandpaper surfaces, grooved plastic cylinders, etc.) [9,10]. This capability to detect small differences in roughness was compared with that of humans using their fingertips. Carvell and Simons [11] also demonstrated that when all but one whisker was removed, the discrimination ability was still present for subjects distinguishing a finely textured surface (50 μm grooved) from a smooth one.

Classical studies in trigeminal ganglion neurons involved head-fixed animals, controlled whisker deflection (*passive deflection*) and recordings of the evoked response [12-14]. These studies distinguished rapidly (RA) and slowly (SA) adapting responses and demonstrated that cells respond differently to different whisker shaft directions and have different velocity sensitivities.

Zucker and Welker [12] investigated the trigeminal cell response in a more naturalistic situation. They artificially stimulated facial muscles to evoke a whisker-like movement in anesthetized rats. They showed that the number of responsive neurons increased when the whisker touched an object. Szwed et al. [15], more recently, used a similar stimulation method for investigating the neuronal response patterns during active touch. They showed that neurons present a large variety of responses to different contact situations (vibrissa sweep, contact, pressure and separation of an object).

A transduction mechanism was recently proposed for texture coding considering the mechanical characteristics of the vibrissae and their location in the whisker pad. The whiskers resonate at different vibration frequencies because of their different lengths [16-18]. Furthermore, Mitchinson *et al *[19] constructed a simulated electro-mechanical model, based on the anatomical and physiological characteristics of the vibrissae system.

During the course of our study an alternative model for texture coding was purposed by Arabzadeh et al [20]. They reported that each sandpaper surface was characterized by a temporal profile of whisker velocity and that this information could be transmitted to the follicle receptors and to the central circuits. They measured whisker vibration during active – artificial – whisking across surfaces and then reproduced the whisker movement by a stimulus playback method for recording the trigeminal and cortex neuronal activity.

The purpose of the present paper was to explore two aspects of texture discrimination. First, we wanted to establish if the primary afferent fiber activity of one vibrissa (gamma) contains information for discriminating textures during active touch. We analyzed the neuronal population response evoked by vibrissa sweeping different materials, and these signals were compared with the control. The active whisking was induced by electrical stimulation of branches of the motor nerve. Wood, metal, acrylic and sandpaper (P1000) were used as the swept surfaces. We polished these surfaces with sandpaper (P1000) to obtain different textures with similar roughness.

Our second objective was to explore the effects of vibrissa pressure on the afferent nerve activity. It was reported that rats use different behavioral strategies to obtain tactile sensory information from objects in the environment. During "exploratory whisking" -the dominant pattern when the animal palpates objects- the frequency ranges between 5–15 Hz but values change between bouts of whisking [21]. When the vibrissa contacts objects that are present only on one side, the movements on the side that make contact have greater amplitudes [22]. Carvell and Simons [9] reported that when the animal palpated a surface, the hair shafts -generally the most caudal whiskers- were continuously bent but to varying degrees. In this paper we test the hypothesis that the rats could use a "optimum" vibrissae pressure for texture discrimination.

We used RMS values to analyze the data in order to relate the amplitude of the afferent nerve discharges to the mechanoreceptor activation. Afferent activity was analyzed in the frequency domain with the Power Spectrum Density (PSD). The PSD was estimated using a Parametric Model that is an alternative to the discrete Fourier transform. In all cases we used ANOVA analysis for comparison.

Our results suggest that the physical properties of the different materials could be transduced during active touch, and varying the pressure against the vibrissae could improve the information acquisition.

## Results

**Figure 1:**
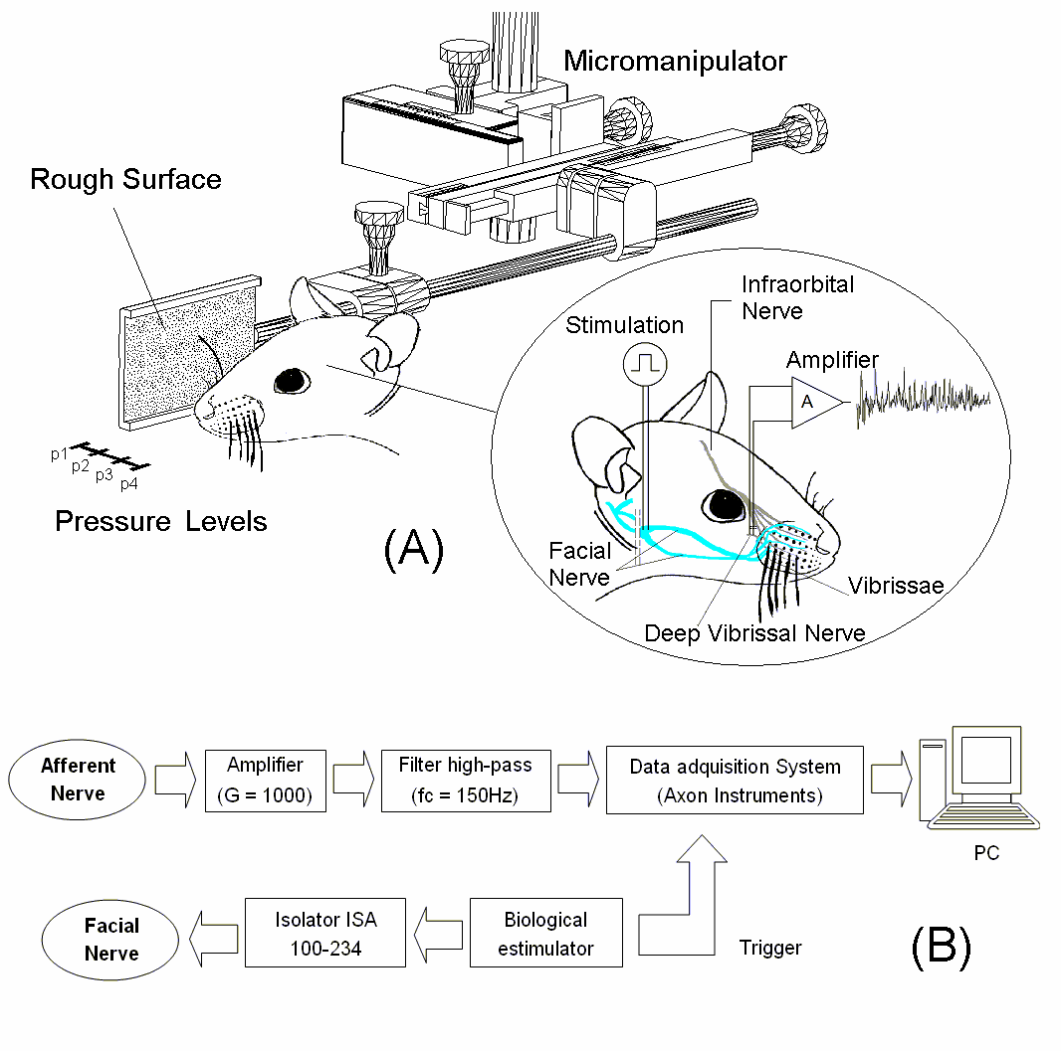
Experimental set up. (A) In the inset form of stimulation of the facial nerve for producing the artificial movement of the vibrissa, and the methodology used to obtain the recordings of the electrical activity in the deep vibrissal nerve is shown. The pressure levels were obtained by using a micromanipulator, and bringing the surfaces closer to the vibrissa by 3 mm each time. (B) Block diagram of the acquisition and stimulation system.

In this study we recorded the multifiber activity from the deep vibrissal nerve of one vibrissa when the hair swept different textures. The whisker movement was induced by electrical stimulation of the facial motor nerve. A diagram of the experimental design is shown in Figure 1. The materials selected for the experiments correspond to surfaces of different texture [see Additional File 1]. We measured only one texture parameter, the roughness, using a Hommel Tester (see Methods). These measurements showed that sandpaper (P1000) had the highest value (4.31 μm) followed by wood (2.52 μm). Metal had the lowest roughness value (0.11 μm), followed by acrylic (0.30 μm) [see Additional File 2].

**Figure 2:**
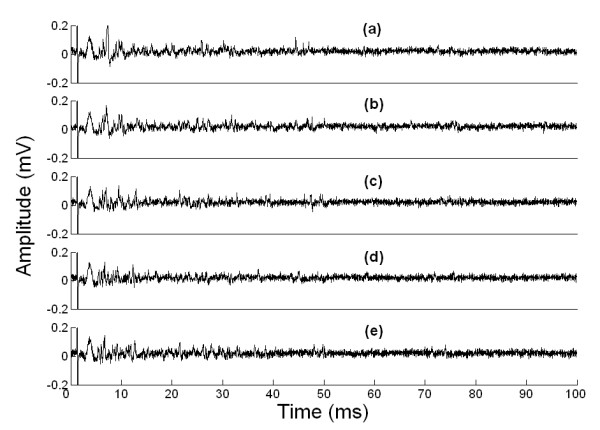
Vibrissal nerve activity recorded when the vibrissa is: (a) sweeping the air (control); (b) sweeping wood; (c) sweeping metal (stainless steel); (d) sweeping acrylic; (e) sweeping sandpaper P1000. All the records show a single vibrissa sweeping each surface at pressure level 1.

Figure 2 shows the afferent discharges when the vibrissa sweeps each surface at pressure level 1. The recordings for pressure levels 2, 3 and 4 are not shown in order to simplify the figure.

**Figure 3:**
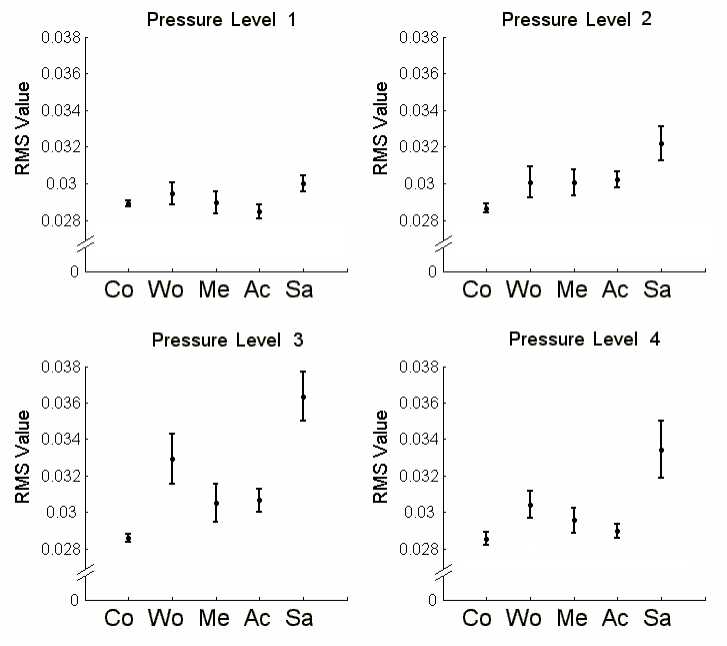
Average RMS values for each sweeping situation and their standard deviations. For each pressure level presented, the RMS value its given for each vibrissa sweeping situation, (Co) sweeping the air; (Wo) sweeping wood; (Me) sweeping metal, (Ac) sweeping acrylic and (Sa) sweeping sandpaper.

Figure 3 shows the RMS values for the four pressure levels and for the different surfaces. Each graph includes the average value and the standard deviation of 50 RMS values. The RMS values increased when the pressure changed from levels 1 to 3, but it behaved irregular for pressure level 4.

Mean control values remained constant and with low deviation in all situations but the materials showed an increased variability (standard deviation). This is particularly clear for sandpaper and wood.

The comparison among treatments (Table 1) for pressure level 1, showed significant differences in all cases except one (control vs. metal; q = 1.075). At pressure level 2, a generalized increment of the average RMS values in all situations was observed. Three comparisons did not show significant differences (wood, metal and acrylic) (Table 1) but sandpaper vs. control showed one of the highest differences (q = 12.333).

The best differenciation was obtained for pressure level 3 (Figure 3). Only metal and acrylic showed similar RMS values and the comparison between them have the lowest value (q = 0.0632) (Table 1). However in all other cases the comparisons showed higher values and the highest was observed between control and sandpaper (q = 12.459).

**Figure 4:**
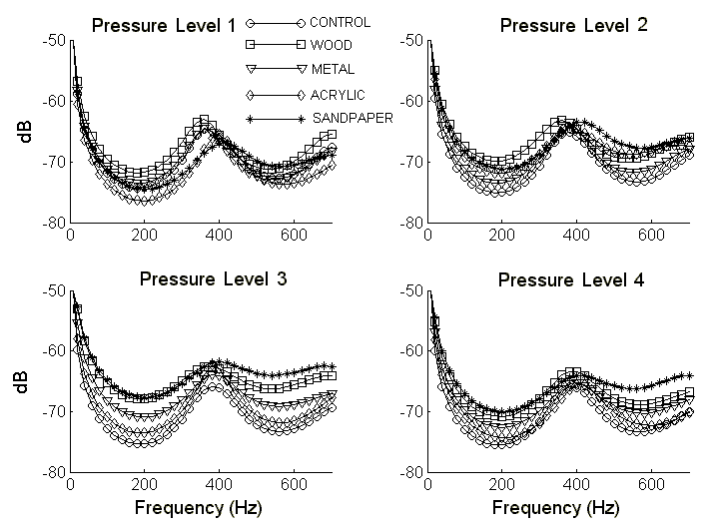
PSD's average of fifty sweeps for each situation.

**Figure 5:**
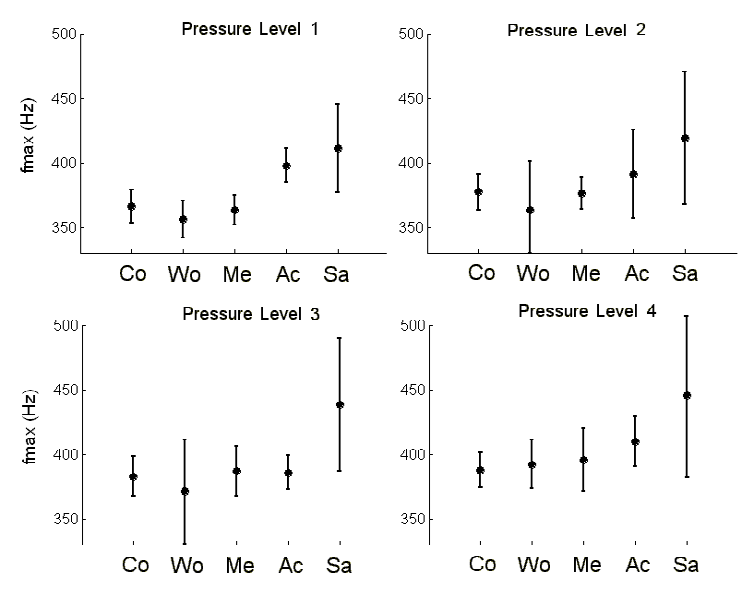
*fmax *values and their respective deviations for each sweeping situation. *fmax *was found within the range of 100 Hz to 600 Hz. Both the average values and the standard deviations were calculated from fifty sweeps for each situation. (Co) sweeping the air; (Wo) sweeping wood; (Me) sweeping metal, (Ac) sweeping acrylic and (Sa) sweeping sandpaper.

The average PSD of the 50 recordings for each surface-pressure combination is shown in Figure 4. Each PSD was represented by *fmax*, calculated within the range of 100 Hz to 600 Hz. Thus, each surface-pressure situation was represented by the average and standard deviation of the 50 fmax values. Figure 5 provides a comprehensive overview of *fmax *changes for each case, and Table 2 shows the statistical results of the comparison.

The most important differences among average PSD's are observed in pressure level 1 (Figure 4). The curves show similar PSD for control, wood and metal, considering their maximum frequency component, and these differ from the average for acrylic and sandpaper. Figure 5 shows that when pressure level increases, *fmax *of the signal is clearly affected by the variance of the *fmax *values. Significant differences in the maximum frequency of the signals, are observed at pressure levels 1 and 2 (Table 2). The comparison at wood vs. acrylic and wood vs. sandpaper had the highest values (q = 7.684 and q = 8.633) for pressure level 1. Metal vs. sandpaper also had a high value (q = 7.400). The values decreased for the remaining pressure levels, except wood vs. sandpaper, which increased slightly (q = 8.823). However, these results couldn t be associated with the roughness of the materials.

## Discussion

Previous studies showed that the rats distinguish surfaces with different roughness [9,11]. Arabzadeh et al. [20] recently described the neural coding properties underlying roughness discrimination by rats. They hypothesized that the kinetics signatures of palpated surfaces define the neuronal firing patterns of first-order and cortex neurons.

In the present work we analyzed the multifiber afferent discharge during active whisking to study texture sensations. Multifiber recordings allowed us to characterize this sensory system considering all neurons activated during a discrimination protocol.

It is known that rats employ strategies to optimize the acquisition of information and these strategies are related to the coordination of motor patterns (frequencies, vibrissa set point, whisker bending, time spent exploring, etc.) [11,23]. Therefore we studied texture discrimination by considering pressure levels as a possible behavioral strategy. A platform with different materials was situated in parallel to the animal axis and perpendicular to the whisker length. To obtain different degrees of pressure, the platform was moved closer to the tip of the whisker (Figure 1) [see Additional File 3] and maintained in this position during the recordings.

The materials used in the present work could be activating the follicle receptors in different ways. Our results suggest that the quantification of the afferent nerve activity (RMS values) could be related to the physical properties of the surfaces, specifically, to the roughness. As roughness increased, the RMS values also increased in almost all cases, suggesting a direct relation (Figure 3). The similar roughness measured for metal and acrylic (0.11 μm and 0.30 μm) could explain the results observed for pressure level 3. The analysis with RMS values suggests that pressure level 3 could be the most effective for improving tactile information acquisition of different textures.

The frequency analysis of the afferent discharge only showed significant differences at pressure level 1 with an important difference observed between wood and sandpaper. This could be due to the differences in their physical characteristics such as spacing between peaks and valleys (average wavelength or spatial frequency) [see Additional File 2].

### Methodological considerations

The vibrissa sweeping was produced by an electrical stimulation of the facial nerve. We used square-wave pulse to simulate the vibrissal whisking at its natural frequency (5 Hz). Whisking movements during artificial whisking may differ from those produced during natural whisking in some details. The vibrissa displacement angle observed in our experiments could differ from that of a behaving rat, as was previously described [9]. However, our stimulus protocol allowed us to record the afferent discharges simultaneously with the muscular activation. In this way we also include the additional information due to active muscle contractions- that could affect sensory receptors during whisking- in our analysis. This was not considered in previous reports about neural coding in texture discrimination.

One important distinction between previous work done using the frequency analysis and the present study is our use of the Parametric Model for PSD analysis.

The Parametric Model is an alternative to discrete Fourier transforms. These avoid problems such as the phenomenon of frequency leakage, frequency resolution, large estimation variance in the case of random signals, implicit assumption of either signal periodicity (Fourier expansion) or signal equal to zero outside the interval of interest (Fourier transform).

## Conclusion

This study investigated the peripheral afferent response of the vibrissal system to tactile stimulation. Our results suggest that physical properties of different materials could be transduced by the trigeminal sensory system of the rats, as are shown by amplitude and frequency component changes.

The relationship between RMS values and material roughness reveal that this physical parameter is better transduced than others. The information in frequency is extremely limited as the pressure level increases and wouldn t be related to the physical properties of the materials used.

The vibrissal pressure variation induced neural activity changes in all cases. Our results reveal that pressure level 3 would be the "optimum" pressure to detect texture differences suggesting a behavioral strategy that improves the information acquisition.

## Methods

### Procedures

Five Wistar adult rats (300 g – 350 g) were used in our experiments. They were anesthetized with urethane (130 mg/Kg) and the temperature of the animal was maintained at 37° by a servo-controlled heating pad.

Surgery consisted of exposing the infraorbital nerve as well as the two branches of the facial nerve (buccal and upper marginal mandibular) on the right side. The motor branches were dissected and transected proximally, and stimulation electrodes were placed on their distal stumps to produce the contraction of the mystacial muscles (Figure 1).

The deep vibrissal nerve innervating one vibrissal follicle (gamma) was identified with the high magnification of a dissecting microscope. We chose the gamma vibrissal nerve because of its easier surgical access. The gamma follicle is located on the last vertical row with three other whiskers (α, β, δ). The corresponding nerve is generally the most dorsally situated in the infraorbital nerve at the zygomatic arch region.

The dissected nerve was transected and a bipolar electrode was placed on it to record the afferent discharge of the corresponding vibrissa (Figure 1). The recording electrodes as well as the nerves were immersed in a mineral oil bath during all recording.

To obtain pressure 1, the tip of the whisker shaft was lightly placed on the surface (the platform surface was in a transverse position with respect to the whisker base). The remaining levels were obtained by moving the surface platform 3 mm closer for each following pressure. This procedure facilitates the whisker curving over the surface during the whisking [see Additional File 3].

All these procedures were done in accordance with the recommendations of the Guide for the Care and Use of Laboratory Animals (National Research Council, NRC).

### Electrophysiological recordings

The experiments consisted in recordings of the multifiber activity of the gamma vibrissal nerve while the vibrissae were sweeping different surfaces. The signal recordings were obtained during active whisking (i.e., simultaneously with the electrical stimulation of the facial nerve and intrinsic muscle contraction).

Since our recordings are simultaneous with the vibrissae muscular activation, the stimulus artifact appears as the first signal followed by the deflection due to the muscle action current. Both deflections were removed before the data were processed and the start of the afferent discharge was estimated at 5 ms from the beginning of the recording. To differentiate the afferent discharge from the noise, we inactivated the follicular nerve by crushing at the end of the experiment. In this way we calculated the time end of the deflection due to the stimulus pulse and the extracellular muscular currents.

Three surfaces (wood, metal, acrylic) were polished using the same grade sandpaper P1000. This procedure allowed us to obtain surfaces with similar roughness and different textures.

The electrical stimulation was induced by a custom-made biological stimulator (developed at the Neuroscience Laboratory, Facultad de Medicina, Universidad Nacional de Tucumán, Argentina). Square-wave pulses (30 μs, 7V supramaximal, 5 Hz) simulated the vibrissal whisking at its natural frequency. An Isolation unit (ISA 100–234, Bioelectric Instruments) was used to isolate the animal from the stimulation device. The afferent nerve signals were filtered with a high-pass filter (fc = 150 Hz).

Each whisking was recorded in a window (sweep) of 100 ms. Fifty windows were obtained for each surface, and another fifty for each control (vibrissa sweeping the air). The controls were inserted between the surface recordings.

The infraorbital nerve signals were digitalized using a data acquisition system, Digidata 1322A, Axon Instruments, at 20 KHz. The parameters of the acquisition were controlled using the software AxoScope. The recordings were acquired immediately after a trigger sent from the electrical stimulator as is shown in a block diagram (Figure 1b).

### Digital processing and statistics

#### RMS value (Root Mean Square)

This parameter allows us to characterize the signal according to its energy content. The energy content was related to the amplitude of the signal in a certain interval of time. For a discrete signal, which consists in N samples equally spaced, the estimate is given by the following equation:

RMS=1N∑k=1N[x(k)]2k=1,2,…,N     (1)
 MathType@MTEF@5@5@+=feaafiart1ev1aaatCvAUfKttLearuWrP9MDH5MBPbIqV92AaeXatLxBI9gBaebbnrfifHhDYfgasaacH8akY=wiFfYdH8Gipec8Eeeu0xXdbba9frFj0=OqFfea0dXdd9vqai=hGuQ8kuc9pgc9s8qqaq=dirpe0xb9q8qiLsFr0=vr0=vr0dc8meaabaqaciaacaGaaeqabaqabeGadaaakeaafaqabeqacaaabaGaemOuaiLaemyta0Kaem4uamLaeyypa0ZaaOaaaeaadaWcaaqaaiabigdaXaqaaiabd6eaobaadaaeWbqaamaadmaabaGaemiEaGNaeiikaGIaem4AaSMaeiykaKcacaGLBbGaayzxaaWaaWbaaSqabeaacqaIYaGmaaaabaGaem4AaSMaeyypa0JaeGymaedabaGaemOta4eaniabggHiLdaaleqaaaGcbaGaee4AaSMaeyypa0JaeGymaeJaeiilaWIaeGOmaiJaeiilaWIaeSOjGSKaeiilaWIaeeOta4eaaiaaxMaacaWLjaWaaeWaaeaacqaIXaqmaiaawIcacaGLPaaaaaa@4EBB@

Where, N, is the number of samples, x(k) is K-th sample of the signal, and RMS is the estimate of the energy.

#### Spectral Estimation – AR modeling

The AR model can be viewed as an all-pole, or infinite-impulse-response (IIR) filter whose current output, s_n_, is a function of both the p most recent outputs, s_n-1_, s_n-2_,..., s_n-p_, and the current input, e_pn_:

sn=s˜n+epn=−∑i=1papi⋅sn−i+epn     (2)
 MathType@MTEF@5@5@+=feaafiart1ev1aaatCvAUfKttLearuWrP9MDH5MBPbIqV92AaeXatLxBI9gBaebbnrfifHhDYfgasaacH8akY=wiFfYdH8Gipec8Eeeu0xXdbba9frFj0=OqFfea0dXdd9vqai=hGuQ8kuc9pgc9s8qqaq=dirpe0xb9q8qiLsFr0=vr0=vr0dc8meaabaqaciaacaGaaeqabaqabeGadaaakeaacqWGZbWCdaWgaaWcbaGaemOBa4gabeaakiabg2da9iqbdohaZzaaiaWaaSbaaSqaaiabd6gaUbqabaGccqGHRaWkcqWGLbqzdaWgaaWcbaGaemiCaaNaemOBa4gabeaakiabg2da9iabgkHiTmaaqahabaGaemyyae2aaSbaaSqaaiabdchaWjabdMgaPbqabaGccqGHflY1cqWGZbWCdaWgaaWcbaGaemOBa4MaeyOeI0IaemyAaKgabeaaaeaacqWGPbqAcqGH9aqpcqaIXaqmaeaacqWGWbaCa0GaeyyeIuoakiabgUcaRiabdwgaLnaaBaaaleaacqWGWbaCcqWGUbGBaeqaaOGaaCzcaiaaxMaadaqadaqaaiabikdaYaGaayjkaiaawMcaaaaa@56CB@

This AR filter can be specified in the frequency domain by taking the z-transform in equation (a). If E(z) and S(z) are the z-transforms of e_p1_, e_p2_,...e_pN _and s_1_, s_2_,...s_N _respectively, then:

*E*(*z*) = *A*(*z*)·*S*(*z*), where A(z)=1+∑i=1papi⋅z−i
 MathType@MTEF@5@5@+=feaafiart1ev1aaatCvAUfKttLearuWrP9MDH5MBPbIqV92AaeXatLxBI9gBaebbnrfifHhDYfgasaacH8akY=wiFfYdH8Gipec8Eeeu0xXdbba9frFj0=OqFfea0dXdd9vqai=hGuQ8kuc9pgc9s8qqaq=dirpe0xb9q8qiLsFr0=vr0=vr0dc8meaabaqaciaacaGaaeqabaqabeGadaaakeaacqWGbbqqcqGGOaakcqWG6bGEcqGGPaqkcqGH9aqpcqaIXaqmcqGHRaWkdaaeWbqaaiabdggaHnaaBaaaleaacqWGWbaCcqWGPbqAaeqaaOGaeyyXICTaemOEaO3aaWbaaSqabeaacqGHsislcqWGPbqAaaaabaGaemyAaKMaeyypa0JaeGymaedabaGaemiCaahaniabggHiLdaaaa@4530@

A−1(z)=S(z)E(z)=1(1+∑i=1papi⋅z−i)     (3)
 MathType@MTEF@5@5@+=feaafiart1ev1aaatCvAUfKttLearuWrP9MDH5MBPbIqV92AaeXatLxBI9gBaebbnrfifHhDYfgasaacH8akY=wiFfYdH8Gipec8Eeeu0xXdbba9frFj0=OqFfea0dXdd9vqai=hGuQ8kuc9pgc9s8qqaq=dirpe0xb9q8qiLsFr0=vr0=vr0dc8meaabaqaciaacaGaaeqabaqabeGadaaakeaacqWGbbqqdaahaaWcbeqaaiabgkHiTiabigdaXaaakiabcIcaOiabdQha6jabcMcaPiabg2da9maalaaabaGaem4uamLaeiikaGIaemOEaONaeiykaKcabaGaemyrauKaeiikaGIaemOEaONaeiykaKcaaiabg2da9maaliaabaGaeGymaedabaWaaeWaaeaacqaIXaqmcqGHRaWkdaaeWbqaaiabdggaHnaaBaaaleaacqWGWbaCcqWGPbqAaeqaaOGaeyyXICTaemOEaO3aaWbaaSqabeaacqGHsislcqWGPbqAaaaabaGaemyAaKMaeyypa0JaeGymaedabaGaemiCaahaniabggHiLdaakiaawIcacaGLPaaaaaGaaCzcaiaaxMaadaqadaqaaiabiodaZaGaayjkaiaawMcaaaaa@5750@

*A*^-1^(*z*) is the AR model's transfer function, usually denoted by *H*(*z*). Its frequency response, *H*(*ω*), is determined by evaluating *H*(*z*) along the unit circle in the z-plane, where *z *= *e*^*jωT *^for a sampling period, T. Furthermore, if *E*(*z*) is a white noise input sequence then its spectrum, *E*(*ω*), will be flat and the spectrum of the output sequence, *S*(*ω*) = *H*(*ω*)·*E*(*ω*), will be equal to *H*(*ω*) scaled by constant, *E*(*ω*) = *E*_*p*_·T. In practice, however, *E*(*z*) only approximates a white noise sequence and so *S*(*ω*) can only be an estimate. This estimate S˜
 MathType@MTEF@5@5@+=feaafiart1ev1aaatCvAUfKttLearuWrP9MDH5MBPbIqV92AaeXatLxBI9gBaebbnrfifHhDYfgasaacH8akY=wiFfYdH8Gipec8Eeeu0xXdbba9frFj0=OqFfea0dXdd9vqai=hGuQ8kuc9pgc9s8qqaq=dirpe0xb9q8qiLsFr0=vr0=vr0dc8meaabaqaciaacaGaaeqabaqabeGadaaakeaacuWGtbWugaacaaaa@2DEA@(*ω*), is given by:

S˜(ω)=EpT|1+∑i=1papi⋅e−ijωT|2     (4)
 MathType@MTEF@5@5@+=feaafiart1ev1aaatCvAUfKttLearuWrP9MDH5MBPbIqV92AaeXatLxBI9gBaebbnrfifHhDYfgasaacH8akY=wiFfYdH8Gipec8Eeeu0xXdbba9frFj0=OqFfea0dXdd9vqai=hGuQ8kuc9pgc9s8qqaq=dirpe0xb9q8qiLsFr0=vr0=vr0dc8meaabaqaciaacaGaaeqabaqabeGadaaakeaacuWGtbWugaacaiabcIcaOGGaciab=L8a3jabcMcaPiabg2da9maaliaabaGaemyrau0aaSbaaSqaaiabdchaWbqabaGccqWGubavaeaadaabdaqaaiabigdaXiabgUcaRmaaqahabaGaemyyae2aaSbaaSqaaiabdchaWjabdMgaPbqabaGccqGHflY1cqWGLbqzdaahaaWcbeqaaiabgkHiTiabdMgaPjabdQgaQjab=L8a3jabdsfaubaaaeaacqWGPbqAcqGH9aqpcqaIXaqmaeaacqWGWbaCa0GaeyyeIuoaaOGaay5bSlaawIa7amaaCaaaleqabaGaeGOmaidaaaaakiaaxMaacaWLjaWaaeWaaeaacqaI0aanaiaawIcacaGLPaaaaaa@55F3@

The assumption on which the AR modeling technique is based can now be rephrased in the frequency domain, where it is assumed that the flat spectrum of the white noise input sequence is "coloured" by the AR model to produce an output spectrum of the desire shape.

### Data processing

Only data between 5 ms and 100 ms were taken into account. This procedure does not include the discharges related to the muscular activation [24], and only the data obtained when the vibrissa was sweeping the air or surfaces were processed.

We consider all sweeps recorded for our analysis. The RMS (Root Mean Square) value was used as an estimator parameter of the signal energy [25]. The Power Spectrum Density (PSD) was calculated by using the Burg parametric estimation method [26]. Both methods were applied to all recordings, obtaining 50 RMS and 50 PSD values for each surface-pressure combination.

The PSD were represented using the maximum frequency (fmax). It was calculated within the range of 100 Hz to 600 Hz.

### Statistics

Statistical analysis was done with one way repeated measure ANOVA on ranks (Friedman) and Dunn's method as a post hoc test (software SigmaStat). RMS and fmax values were compared for recordings with the same level of pressure.

Data processing, RMS, PSD and fmax calculations were carried out by using MATLAB.

### Textures measurements

Surface texture is not a measurable quantity; it is not possible to assign a unique "texture" value to every different surface. However, it is possible to measure some of the intrinsic characteristics, or parameters, of surface texture. The International Standards BS.1134 and ISO 468 characterize the textures by means of "surface textures parameters". The surface textures parameters defined by the standard are the following:

1. Roughness, is a measure of the vertical characteristics of the surface.

2. Skewness, It is a non-dimensional parameter, which measures the symmetry of the surface about the mean plane.

3. Sharpness, the sharpness of the surface is defined by the kurtosis, another non-dimensional surface texture parameter.

4. Average wavelength (λ), is a measure of the spacing between peaks and valleys, taking into account their relative amplitudes and individual spatial frequency. The generalization of λ is difficult because its definition necessarily implies a direction.

We measured the materials roughness using a Hommel Tester T1000 (Hommel Werke) and used the *Ra *parameter (arithmetical deviation of the assessed profile) as a roughness estimation (International Standards BS.1134 and ISO 468) [see Additional File 2].

## Authors' contributions

ALA carried out the surgery and the electrophysiological studies, participated in the data interpretation and drafted the manuscript. FDF carried out the digital processing and statistics and participated in the design of the experiments and writing. CJF assisted in digital processing and data interpretation. EED conceived the study.

All authors read and approved the manuscript.

## Supplementary Material

Additional File 1 **Surfaces Pictures**. Photographs of the surfaces used in this paper. Click here for file

Additional File 2 **Roughness Measures**. Surfaces roughness measurements obtained using a Hommel Tester T1000 (Hommel Werke). The Ra parameter (arithmetical deviation of the assessed profile) is a roughness estimation (International Standards BS.1134 and ISO 468). Click here for file

Additional File 3 **Pressure levels**. This figure shows a scheme of the gamma vibrissal shaft and the different pressure levels obtained approaching the surfaces. At pressure level 1, the vibrissa remains in contact with the surface without undergoing deformation. Click here for file

## Figures and Tables

**Table 1:** Results of the multiple comparisons applied to RMS values (Dunn Method) The test computes statistic Q, the number of rank sums, and shows whether P < 0.05 or not, for the pair being compared. P is the probability that the null hypothesis may be rejected and so concludes that there are differences between treatments. *Diff of ranks *is the difference in the rank sum orders being compared. The rank sums are a measure of the difference between two treatments.

	**Pressure level 1**			**Pressure level 2**		
**Comparison**	**Diff of Ranks**	**Q**	**P < 0.05**	**Diff of Ranks**	**Q**	**P < 0.05**

Control vs Wood	1.500	4.743	Yes	1.800	5.692	Yes
Control vs Metal	0.340	1.075	No	1.900	6.008	Yes
Control vs Acrylic	0.960	3.036	Yes	2.300	7.273	Yes
Control vs Sandpaper	2.520	7.969	Yes	3.900	12.333	Yes
Wood vs Metal	1.160	3.668	Yes	0.100	0.316	No
Wood vs Acrylic	2.460	7.779	Yes	0.500	1.581	No
Wood vs Sandpaper	1.020	3.226	Yes	2.100	6.641	Yes
Metal vs Acrylic	1.300	4.111	Yes	0.400	1.265	No
Metal vs Sandpaper	2.180	6.894	Yes	2.000	6.325	Yes
Acrylic vs Sandpaper	3.480	11.005	Yes	1.600	5.060	Yes

	**Pressure level 3**			**Pressure level 4**		

**Comparison**	**Diff of Ranks**	**Q**	**P < 0.05**	**Diff of Ranks**	**Q**	**P < 0.05**

Control vs Wood	2.820	8.918	Yes	2.500	7.906	Yes
Control vs Metal	1.580	4.996	Yes	1.620	5.123	Yes
Control vs Acrylic	1.560	4.933	Yes	0.700	2.214	No
Control vs Sandpaper	3.940	12.459	Yes	3.680	11.637	Yes
Wood vs Metal	1.240	3.921	Yes	0.880	2.783	No
Wood vs Acrylic	1.260	3.984	Yes	1.800	5.692	Yes
Wood vs Sandpaper	1.120	3.542	Yes	1.180	3.731	Yes
Metal vs Acrylic	0.020	0.0632	No	0.920	2.909	Yes
Metal vs Sandpaper	2.360	7.463	Yes	2.060	6.514	Yes
Acrylic vs Sandpaper	2.380	7.526	Yes	2.980	9.424	Yes

**Table 2:** Results of multiple comparisons applied to maximum frequency values. *fmax *(Dunn method). The test computes statistic *Q*. the number of rank sums. and shows whether P < 0.05 or not for the pair being compared. P is the probability that the null hypothesis may be rejected and so concludes that there are differences between treatments. *Diff of ranks *is the difference in the rank sum orders being compared. The *rank sums *are a measure of the difference between two treatments.

	**Pressure level 1**			**Pressure level 2**		
**Comparison**	**Diff of Ranks**	**Q**	**P < 0.05**	**Diff of Ranks**	**Q**	**P < 0.05**

Control vs Wood	0.600	1.897	No	1.120	3.542	Yes
Control vs Metal	0.210	0.664	No	0.140	0.443	No
Control vs Acrylic	1.830	5.787	Yes	0.740	2.340	No
Control vs Sandpaper	2.130	6.736	Yes	1.670	5.281	Yes
Wood vs Metal	0.390	1.233	No	0.980	3.099	Yes
Wood vs Acrylic	2.430	7.684	Yes	1.860	5.882	Yes
Wood vs Sandpaper	2.730	8.633	Yes	2.790	8.823	Yes
Metal vs Acrylic	2.040	6.451	Yes	0.880	2.783	No
Metal vs Sandpaper	2.340	7.400	Yes	1.810	5.724	Yes
Acrylic vs Sandpaper	0.300	0.949	No	0.930	2.941	Yes

	**Pressure level 3**			**Pressure level 4**		

**Comparison**	**Diff of Ranks**	**Q**	**P < 0.05**	**Diff of Ranks**	**Q**	**P < 0.05**

Control vs Wood	0.600	1.897	No	0.400	1.265	No
Control vs Metal	0.210	0.664	No	0.450	1.423	No
Control vs Acrylic	0.040	0.126	No	1.380	4.364	Yes
Control vs Sandpaper	1.200	3.795	Yes	1.570	4.965	Yes
Wood vs Metal	0.810	2.561	No	0.050	0.158	No
Wood vs Acrylic	0.640	2.024	No	0.980	3.099	Yes
Wood vs Sandpaper	1.800	5.692	Yes	1.170	3.700	Yes
Metal vs Acrylic	0.170	0.538	No	0.930	2.941	Yes
Metal vs Sandpaper	0.990	3.131	Yes	1.120	3.542	Yes
Acrylic vs Sandpaper	1.160	3.668	Yes	0.190	0.601	No
