# Supplementary figures and images for: Texture discrimination and multi-unit recording in the rat vibrissal nerve
Source: BMC Neurosci. 2006 May 23;7:42. doi: 10.1186/1471-2202-7-42 (PMC1525197; doi:10.1186/1471-2202-7-42)

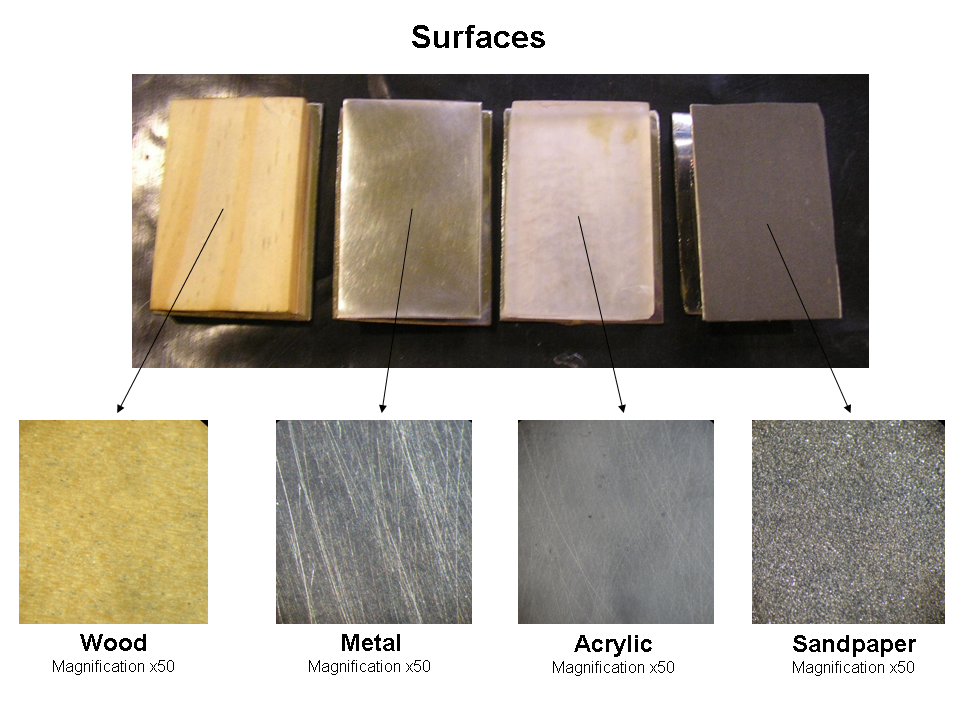

Supplement: Additional File 1 — Surfaces Pictures. Photographs of the surfaces used in this paper. [file 1471-2202-7-42-S1.tiff]

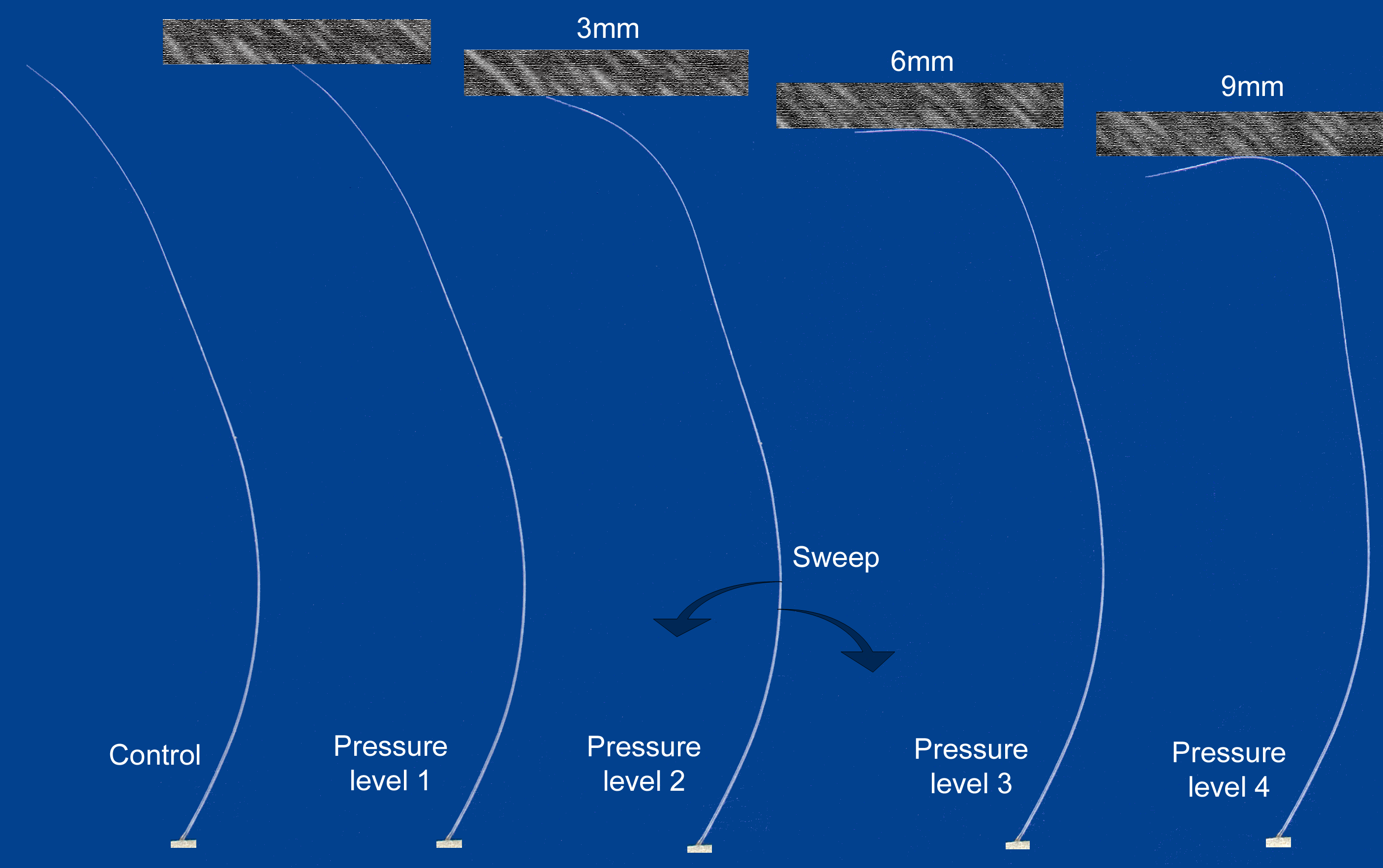

Supplement: Additional File 3 — Pressure levels. This figure shows a scheme of the gamma vibrissal shaft and the different pressure levels obtained approaching the surfaces. At pressure level 1, the vibrissa remains in contact with the surface without undergoing deformation. [file 1471-2202-7-42-S3.tiff]
